# Ultrafine-Grained Ti-31Mo-Type Composites with HA and Ag, Ta_2_O_5_ or CeO_2_ Addition for Implant Applications

**DOI:** 10.3390/ma14030644

**Published:** 2021-01-30

**Authors:** Patrycja Sochacka, Mieczyslawa U. Jurczyk, Kamil Kowalski, Przemyslaw K. Wirstlein, Mieczyslaw Jurczyk

**Affiliations:** 1 Institute of Materials Science and Engineering, Poznan University of Technology, Jana Pawła II 24, 61-138 Poznan, Poland; kamil.kowalski@put.poznan.pl (K.K.); mieczyslaw.jurczyk@put.poznan.pl (M.J.); 2 Division of Mother’s and Child’s Health, Poznan University of Medical Sciences, Polna 33, 60-535 Poznan, Poland; mjur@poczta.onet.pl; 3 Department of Gynaecology and Obstetrics, Division of Reproduction, Poznan University of Medical Sciences, Polna 33, 60-535 Poznan, Poland; abys@wp.pl

**Keywords:** Ti31Mo alloy, hydroxyapatite, biomaterials, ultrafine grain, metal matrix composites, cell proliferation, MTS assay

## Abstract

Ultrafine-grained Ti31Mo alloy and Ti31Mo5HA, Ti31Mo5HA-Ag (or Ta_2_O_5_, CeO_2_) composites with a grain size of approximately 2 μm were produced by the application of mechanical alloying and powder metallurgy. Additionally, the surface of the Ti31Mo alloy was modified. In the first stage, the specimens were immersed in 5M NaOH for 24 h at 60 °C. In the second stage, hydroxyapatite (HA) was deposited on the sample surface. The cathodic deposition at −5 V vs. open circuit potential (OCP) in the electrolyte containing 0.25M CaNa_2_-EDTA (di-calcium ethylenediaminetetraacetic acid), 0.25M K_2_HPO_4_ in 1M NaOH at 120 °C for 2 h was applied. The bulk Ti31Mo alloy is a single β-type phase. In the alkali-modified surface titanium oxide, Ti_3_O is formed. After hydrothermal treatment, the surface layer mostly consists of the Ca_10_(PO_4_)_6_(OH)_2_ (81.23%) with about 19% content of CaHPO_4_·2H_2_O. Using optical profiler, roughness 2D surface topography parameters were estimated. The in vitro cytocompatibility of synthesized materials was studied. The cell lines of normal human osteoblasts (NHost) and human periodontal ligament fibroblasts (HPdLF) was conducted in the presence of tested biomaterials. Ultrafine-grained Ti-based composites altered with HA and Ag, Ta_2_O_5_ or CeO_2_ have superior biocompatibility than the microcrystalline Ti metal. NHost and HPdLF cells in the contact with the synthesized biomaterial showed stable proliferation activity. Biocompatibility tests carried out indicate that the ultrafine-grained Ti31Mo5HA composites with Ag, Ta_2_O_5,_ or CeO_2_ could be a good candidate for implant applications.

## 1. Introduction

Stainless steel, cobalt alloys, titanium, and titanium-base alloys are applied as implant materials. These cannot be substituted by ceramics or polymers because of their high strength and toughness [1]. Stainless steel and Co–Cr–Mo due to high Young modulus in comparison to that of bone tend to fail after long-term use.

In the production of dental and orthopedic implants titanium and its alloys are used [2]. These biomaterials have interesting properties: low density (4.5 g/cm^3^), low Young modulus (about 100 GPa), high tensile strength (240 MPa), and due to the passive titanium oxide film (TiO_2_) have acceptable corrosion properties [3]. On, the other hand, titanium and titanium alloys, due to low hardness, have poor tribological properties [4]. Additionally, some research reports signifying metal release and corrosion in vivo [5,6].

The aim of the current study is directed at improving the physicochemical and mechanical properties as well as biocompatibility of Ti-based systems through crystal structure evolution (α → β) and new microstructure formation (micro → nano or ultrafine) via its chemical composition modification and processing method, respectively [4,5,7,8,9,10,11,12,13,14,15,16,17,18,19]. β-type titanium alloys are interesting biomaterials for innovative implantable medical devices. Recently studies on TiMo, TiNb, TiZrNb, TiNbHf, TiNbZrTa, TiNbZrTaSiFe have confirmed their interesting properties for future application in medicine [9,13,14,15,16,17,19,20,21,22,23,24,25]. It is important to note, that the cytotoxicity of Mo, Zr, and Nb is lower than that for commercial purity Ti [26].

The latest studies have proven that Ti–Mo alloys open new possibilities for advanced medical implants [13,16,17]. The solubility limits of molybdenum in titanium is 8 wt. % [27]. The phase evolutions and properties of Ti–Mo (10 ≤ x ≤ 35) alloys were studied [16]. Additionally, the influence of the preparation method on transitions (α → β) as well as on the microstructure, mechanical, corrosion, and surface wettability properties was investigated, as well [17]. It is possible to obtain Ti–Mo alloys with high β-phase content and also low porosity by using hot pressing (HP) at low temperature (800 °C/5 min) compared to cold pressing and sintering (800 °C/0.5 h).

One possibility to enhance the mechanical, corrosion, and biological properties of implant materials, except the chemical composition modification, is the microstructure control via severe plastic deformation (SPD) [28,29] or mechanical alloying (MA) processing methods [11,12,15,16,17,18,19]. Published results proved that the nano- or ultrafine grain microstructure of titanium and its alloys improved the mechanical properties as well as the biocompatibility [18,19,29,30,31,32].

To change the biological properties of titanium alloys, composites can be synthesized that combine good mechanical properties of titanium and the excellent biocompatibility and bioactivity of ceramics (hydroxyapatite (HA), 45S5 Bioglass) [33]. Bioceramic–titanium composites will have practical applications in medicine and can replace titanium alloys with a ceramic coating. It is well known that ceramic coating improves the surface bioactivity, however, it often falls off due to the poor ceramic/metal interface bonding [11,19,34].

Biomaterials with nano- or ultrafine- grains offer an interesting property for new products in medical applications [4,10,15,16,17,18,19,28,29,30,31,32,35]. Our previous studies confirmed that Ti or Ni-free 316L stainless steel—hydroxyapatite composites exhibit superior properties due to the nanostructure. [10,36]. The mechanical properties and corrosion resistance on bulk Ti-*x* wt. % 45S5 Bioglass nanocomposites (*x* = 0, 3, 10, and 20) were investigated [10]. For example, the bulk Ti-10 wt. % 45S5 Bioglass composite in comparison to pure titanium is more corrosion resistant and twice as harder. Ti-10 wt. % 45S5 Bioglass scaffold shows an enhanced property for dental implant applications. These composites show better cytocompatibility in comparison with microcrystalline commercial purity titanium.

Another example of materials for potential applications in dentistry and medicine are independently synthesized bulk metal matrix nanocomposites (MMNC) based on titanium and boron [12]. Novel in situ bionanomaterials MMNC based on Ti-B obtained in the processes of mechanical synthesis and powder metallurgy show new properties compared to the microcrystalline counterpart. The combination of their unique structure with good mechanical properties, as well as cell viability and cytological compatibility depending on the processing conditions favor the nanoscale range of results of the Ti-TiB. [12].

Recently, ultrafine-grained Ti-Zr-Nb type composites with 45S5 Bioglass and Ag, Cu, or Zn metals have synthesized, as well [19]. Higher biocompatibility than the reference material (microcrystalline Ti) was observed. Ti23Zr25Nb-9BG composite has interesting mechanical properties. An elastic modulus equals 45 GPa, which is lower than the E modulus for Ti23Zr25 samples with 70% porosity (55 GPa).

In this paper in vitro cytocompatibility of Ti-31 Mo alloy and Ti31Mo5HA, Ti31Mo5HA-Ag (or Ta_2_O_5_, CeO_2_) biocomposites were investigated and compared with commercial purity (CP) Ti. Additionally, the surface of the basic Ti31Mo alloy was modified. The biomaterials were tested on the cell lines of normal human osteoblasts (NHost) and human periodontal ligament fibroblasts (HPdLF). The studies aimed to confirm superior biocompatibility of ultrafine-grained Ti-based composites altered with HA and Ag, Ta_2_O_5,_ or CeO_2_.

## 2. Materials and Methods

### 2.1. Sample Preparation

Details on sample synthesis are available in our recent papers [16,18]. The ultrafine-grained Ti31Mo-type samples (diameter—6 mm; height—3 mm) were synthesized by MA and powder metallurgy. High-purity powder precursors from Alfa-Aesar, Heysham, Lancashire; United Kingdom (Ti), Sigma-Aldrich, St. Louis, MO, USA), (Mo, HA, Ag, Ta_2_O_5_, and CeO_2_) were used. In the first stage, the powders were ground in SPEX 8000 Mixer Mill (SPEX SamplePrep, Metuchen, NJ, USA) for 39 h at room temperature and the ball to powder ratio (BPR) was 10:1. Then, the green compacts were obtained by uniaxial pressing (600 MPa) and finally were sintered at 800 °C for 0.5 h in an argon atmosphere.

Polishing, then washing with distilled water, rinsing and degreasing ultrasonically in ethanol, and finally, air-drying were carried out to prepare the surface of the samples for surface treatment. To obtain an oxide layer on the surface the samples were immersed in 5M NaOH (Poch S.A., Gliwice, Poland) for 24 h in a vessel heated to 60 °C in a furnace. After the alkali treatment specimens were washed with distilled water and ethanol. Finally, the hydroxyapatite was deposited on the surface. Aqueous electrolyte containing 0.25M CaNa_2_-EDTA (Sigma-Aldrich, St. Louis, MO, USA), 0.25M K_2_HPO_4_ (Alfa-Aesar, Heysham, Lancashire; United Kingdom) in 1M NaOH (Poch S.A., Gliwice, Poland) at 120 °C for 2 h was applied.

For this study, the synthesized Ti-31Mo—materials were labeled as follows:

- ultrafine-grained Ti31Mo alloy—Ti31Mo
- ultrafine-grained Ti31Mo alloy after NaOH 60 °C/24 h oxidation—Ti31Mo (Ox)
- ultrafine-grained Ti31Mo alloy after hydrothermal treatment—Ti31Mo (HT)
- ultrafine-grained Ti31Mo-5 wt. % HA composite—Ti31Mo5HA
- ultrafine-grained Ti31Mo-5 wt. % HA-1 wt. % Ag composite—Ti31Mo5HA1Ag
- ultrafine-grained Ti31Mo-5 wt. % HA-2 wt. % CeO_2_ composite—Ti31Mo5HA2CeO_2_
- ultrafine-grained Ti31Mo-5wt. % HA-2 wt. % Ta_2_O_5_ composite—Ti31Mo5HA2Ta_2_O_5_

### 2.2. Materials Characterization

The crystal structure was studied by the application of Panalytical Empyrean equipment with copper radiation; l = 1.54 Å (Almelo, The Netherlands). Additionally, the Rietveld approach was used on the Maud software (Luca Lutterotti, University of Trento, Trento, Italy) for the crystal data estimation and phase quantitative analysis [11,12,15,16,17,18,19]. The applied estimation involved the simulation of the diffraction patterns based on the structural models for Ti(β) (ref. code 01-074-7075), Ti_3_O (ref. code 01-073-1117), Ca_10_(PO_4_)_6_(OH)_2_ (ref. code 04-010-6315), and CaHPO_4_·2H_2_O (ref. code 01-072-1240). The chemical compositions and microstructure of the studied alloy and composites were investigated by the application of a scanning electron microscope (SEM, VEGA 5135, and Mira 3, Tescan, Brno, Czech Republic) with an energy-dispersive spectrometer (EDS, PTG Prison Avalon). T8000 Profiler (Hommel–Etamic, Villingen-Schwenningen, Germany) was applied to analyze the surface morphology of the samples. The EVOVIS software (Hommel-Etamic, Villingen-Schwenningen, Germany) was applied to analyze the obtained profiles. The arithmetic mean roughness (μm)—R_a,_ the maximum height of the profile μm)—R_t_, 10-point mean roughness (μm)—R_z_ was estimated. The weight loss (W) of the samples, after immersion in them for 7 days in the Ringer solution environment, was measured to evaluate the corrosion resistance of synthesized biomaterials. Digital camera Kruss-DSA25 (KRÜSS GmbH, Hamburg, Germany) and Kruss-Advanced 1.5 software (KRÜSS GmbH, Hamburg, Germany) and ellipse fitting method were used to determine the contact angles for diiodomethane and glycerol at 23 °C [37]. The application of Owens, Wendt, Rabel, and Kaelble method allowed to establish the surface free energy (SFE) [38,39].

### 2.3. In Vitro Evaluation

The in vitro cytocompatibility investigations were done under standard conditions in 96 well culture dishes in the Heraeus BB16 incubator (Heraeus Instruments GmbH Bereich Termotech, Hanau, Germany) at 37 °C temperature, in an atmosphere of 5% CO_2,_ and humidity level of 95%. The discs of Ti31Mo—type materials, as well as microcrystalline titanium, were sterilized by immersion in 70% of the EtOH dilution and drying in a laminar flow hood with the ultraviolet (UV) sterilization of each side of the insert for 12 h.

Normal human osteoblasts (NHost., CC-2538) and human periodontal ligament fibroblasts (HPdLF, CC-7049) were ordered together with a dedicated set of breeding media, respectively: CC-3207 OGM Osteoblast Growth BulletKit (CC-3208+CC-4193) and CC-3205 SCGM Stromal Bullet CellKit (CC-3204+CC-4181) at LONZA Group Ltd. (Morristown, NJ, USA). More details related to cell line preparation with the conditioning of breeding media are available in our recently published paper [19].

To assess the number of cell proliferation, viability, and cytotoxicity the CellTiter 96^®^ AQueous Non-Radioactive Cell Proliferation Assay (MTS) (Promega, Madison, WI, USA) was applied [12,19,32,35]. The MTS assay protocol is based on the reduction of the MTS tetrazolium compound by viable cells to generate a colored, soluble formazan product the quantity of which was measured spectrophotometrically (λ = 490 nm) in the ELISA plate reader, (MRX Dynex, Chantilly, VA, USA). Microcrystalline titanium (Ti) was applied as reference material of the cell growth in the conditioned media of the composite Ti31Mo-type samples. The cells were grown in triplicates for 24, 72, and 120 h in each cell type and test materials. The MTS test results were averaged for each type of cells and conditioned medium. The relative viability of the cells (RVC) was calculated based on the value of absorbance [19].

Photographic documentation of the cell cultures was conducted in conditioned media in 24 well dishes, on sterile 13 mm cover slides. The photographic documentation was made in the magnification of 150 × by the application of a Nikon digital camera (Nikon, Minato-ku, Tokyo, Japan).

## 3. Results

### 3.1. Crystal Structure, Phase Contents, the Morphology

The uniaxial pressing of Ti31Mo type materials leads to the formation of green compacts. These were sintered at 800 °C for 0.5 h. The Ti31Mo alloy showed Ti(β)-type phase (a = 3.2433 Å). In the Ti31Mo5HA composite, mainly β-type phases (61.1%), as well as the α-Ti (24.3%) and Ti_3_P (14.6%), were observed. The presence of the Ti_3_P phase confirms the decomposition of HA during the MA process. More details related to the HA content on the crystal structure of Ti-31Mo alloy can be found in our previous paper [18].

**Figure 1:**
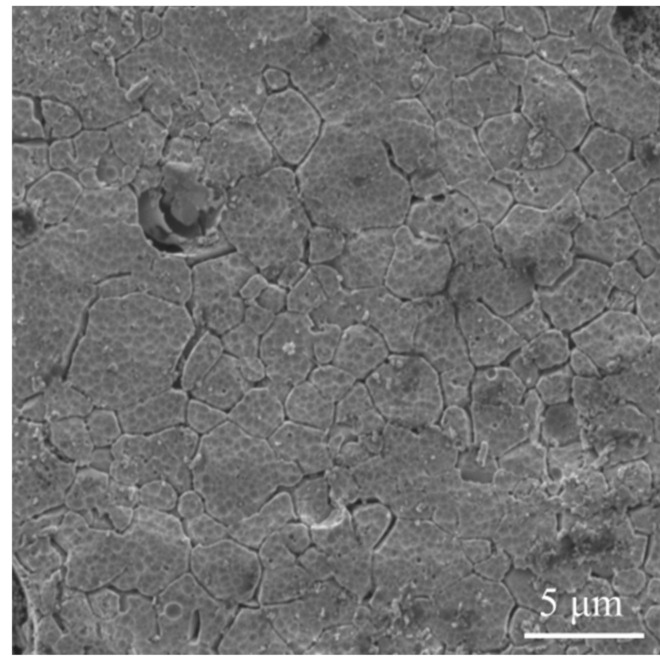
Scanning electron microscopy (SEM) micrograph of the bulk ultrafine-grained Ti31Mo alloy.

In bulk Ti31Mo5HA composite with 1 wt. % Ag, 2 wt. % Ta_2_O_5_ and 2 wt.% CeO_2_ the multiphase material were observed: regular phases Ti_0.67_Mo_0.33_, Ti_0.75_Mo_0.25_, β-Ti in total amounts 65.4, 54.6, and 74.5, respectively, and hexagonal α-Ti forms as also Ti_3_P, Ti_4_P_3_ related to HA addition. The SEM micrograph of the bulk ultrafine-grained Ti31Mo alloy is shown in Figure 1. The average grains of about 2 μm can be seen for this sample. The average grain size is about 150–170 μm in microcrystalline Ti.

**Figure 2:**
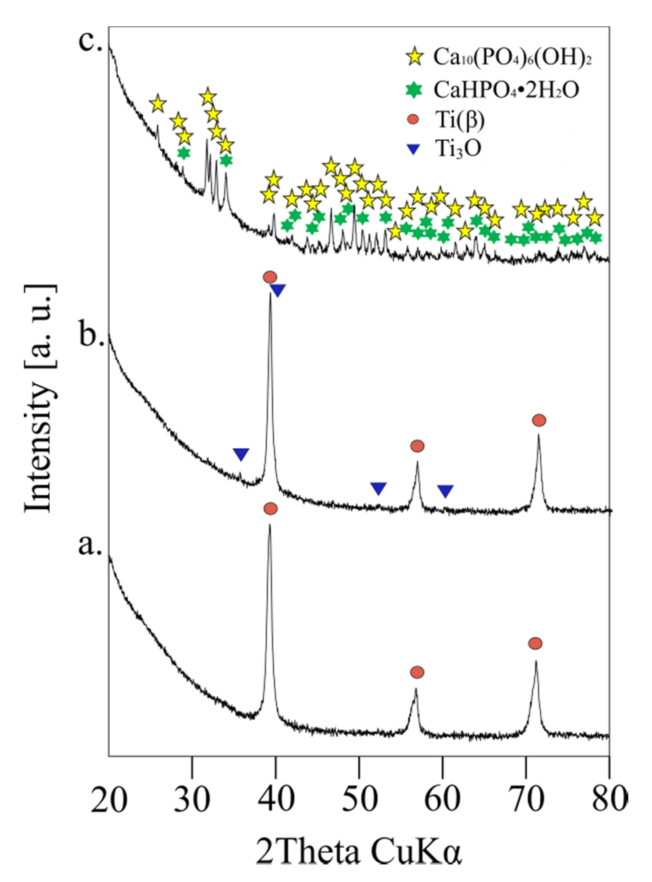
Ti31Mo alloy before modification (**a**), after NaOH 60 °C/24 h (**b**), and hydrothermal treatment (**c**).

Figure 2 shows X-ray diffraction (XRD) results of ultrafine-grained Ti31Mo alloy before (a), after NaOH 60 °C/24 h (b), and hydrothermal treatments (c). The bulk Ti31Mo alloy is a single β-type phase. In the alkali modified (5M NaOH for 24 h at 60 °C) surface titanium oxide, Ti_3_O, is formed. Its content equals 4.93%. After hydrothermal treatment, the surface layer mostly consists of the Ca_10_(PO_4_)_6_(OH)_2_ (81.23%) with about 19% content of CaHPO_4_·2H_2_O (Table 1). Due to their bioactive properties, these ceramics are useful in bone surgery. Additionally, they are non-toxic and non-allergic.

**Figure 3:**
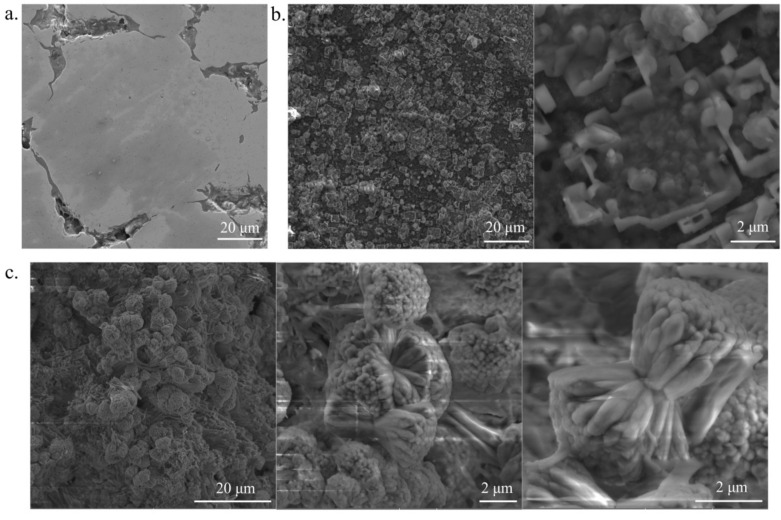
SEM micrographs for Ti31Mo before modification (**a**), after NaOH 60 °C/24 h (**b**), and hydrothermal treatment (**c**).

**Figure 4:**
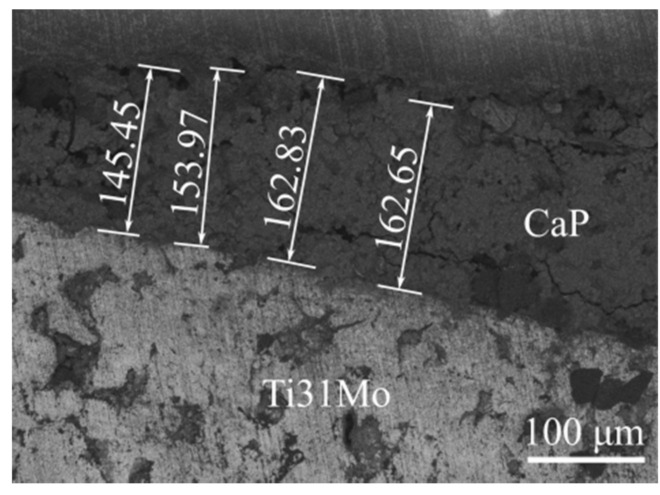
Cross-section view of CaP surface layer for Ti31Mo after hydrothermal treatment.

In the alkali and hydrothermally treated Ti31Mo alloy, the hydroxyapatite was deposited. Porous Ca-P protrusions in cauliflower-like shape are visible (Figure 3). The morphology, size, and structural organization of HA particles could be controlled by changing the temperature and time during the HT process. The osteoblast cells will be grown on that porous Ca-P layer. Figure 4 shows a cross-section view of the surface layer with a thickness close to 155 μm.

**Figure 5:**
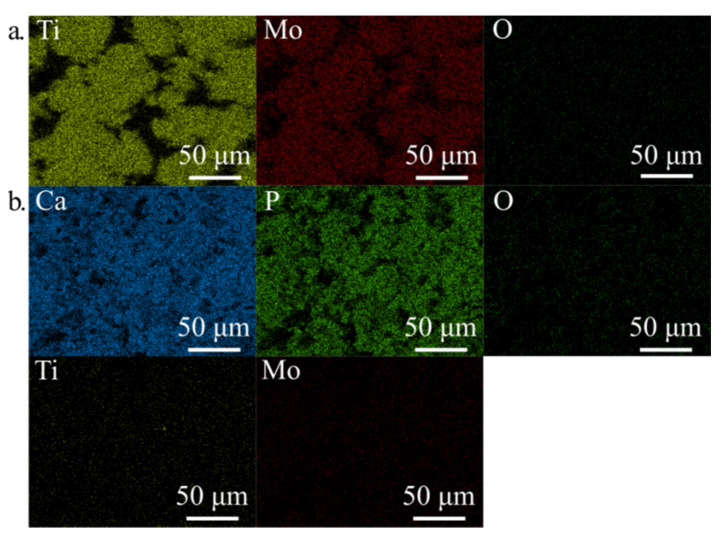
EDS mapping for Ti31Mo after NaOH 60 °C/24 h (**a**) and hydrothermal treatment (**b**).

The EDS analysis confirmed the XRD analysis and proved the content of both Ca and P on the surface after HT treatment (Table 2, Figure 5). After immersion in 5M NaOH at 60 °C for 24 h, the average sodium content was equal to 2.22 wt. % due to the presence of the sodium titanate [40] which can positively influence the nucleation and growth of the surface layer. EDS analysis of the deposited Ca-P layer (Figure 3 and Figure 4) confirmed the formation of hydroxyapatite (HA), which was similar to the human hard tissues in morphology and composition. An important property of HA is its stability in the body fluids in comparison to other calcium phosphates.

### 3.2. Surface Properties

**Figure 6:**
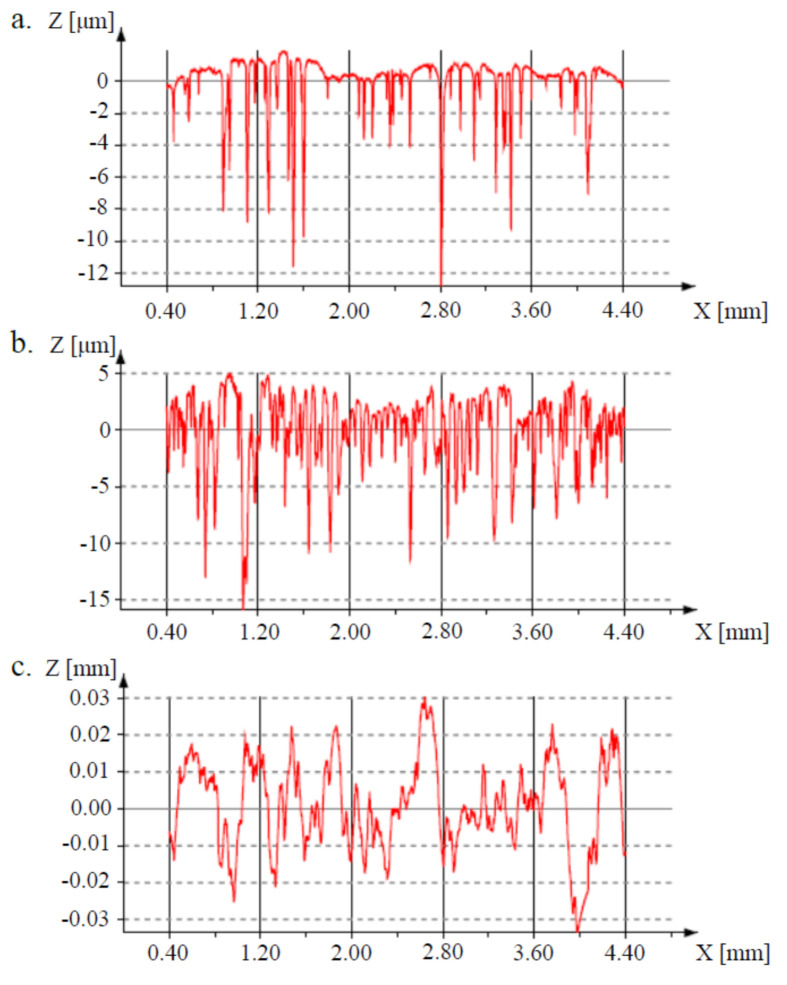
The surface roughness of the Ti31Mo before modification (**a**), after NaOH 60 °C/24 h (**b**), and hydrothermal treatment (**c**).

Figure 6 shows X-profiles, at the different processing stages, of the bulk Ti31Mo alloy. Surface roughness is a leading property of the implant during the osseointegration. The proliferation of cells can be supported also by nano-topography [29,31,41]. The bulk ultrafine-grained Ti31Mo alloy had Ra, Rt, and Rz values of approximately 1.04, 14.55, and 10.59 μm, respectively (Table 3). After NaOH 60 °C/24 h (b) and hydrothermal treatments this alloy surface had an average Ra, Rt, and Rz values in the range of 2–9, 22–62, and 16–45 μm, respectively. Large pores formed on the surface of the sample during the two steep of treatment aid proliferation. The highly developed surface morphology obtained after additional Ca-P deposition facilitates the colonization by pathogenic microorganisms [42,43,44,45,46].

### 3.3. Corrosion and Surface Wetting Properties

Plasma-spraying, grit blasting, acid etching, anodization, or calcium phosphate coatings are methods used to reduce the corrosion rate of titanium alloys in simulated body fluids [8,11,47,48,49,50]. In this study, the corrosion resistance was evaluated by the weight loss (W) of the samples after immersion in the Ringer solution environment. In the case of Ti31Mo alloy after HT treatment W equals 0.0150 mg/day (Table 4). The alkali treatment led only to a decrease in weight loss, which was caused by the Na_2_Ti_2_O_4_(OH)_2_ and Ti_3_O formation. The same positive effect was observed previously in the case of mechanically alloyed and sintered titanium-hydroxyapatite nanocomposites and other Ti-based metallic biomaterials [51,52,53]. Better corrosion resistance is possible with Ti-Bioglass(45S5)-Ag composites due to the rutile layer on the surface [10].

The surface contact angles and free energy in diiodomethane and glycerol were significantly improved for an electrochemically etched and deposited sample (Table 4). Surface free energy was measured to be about 43 mN/m after NaOH 60 °C/24 h treatment. On the other hand, contact angles were decreased to about 31°, after hydrothermal treatment, in the case of glycerol. As a result, the materials after surface treatment were more hydrophilic, which promotes the growth of bone tissue. This surface had a positive effect on the absorption, adhesion, and cell proliferation activity [54].

### 3.4. Biocompatibility Studies

An in vitro test is a method to test for the toxicity of a biomaterial [54,55]. In our study, we tested Normal Human Osteoblasts and Human Periodontal Ligament Fibroblasts. The MTS [3-(4,5-dimethylthiazol-2-yl)-5-(3-carboxymethoxyphenyl)-2-(4-sulfophenyl)-2H -tetrazolum] assay was used to assess cell proliferation in a conditioned medium. The tetrazolium salts were reduced by viable cells to formazan products that are directly soluble in the cell culture medium. The quantity of formazan product was measured. Microcrystalline titanium (Ti) was applied as reference material for the cell growth of the composite Ti31Mo-type samples.

**Figure 7:**
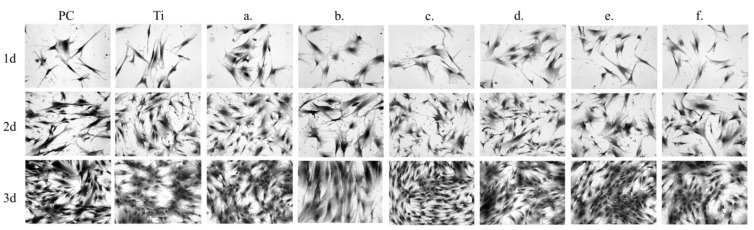
Morphology of the normal human osteoblast (NHost) cells cultured for a different time: 24 h, 3 days, and 5 days: Ti31Mo (**a**), Ti31Mo after hydrothermal treatment (HT) (**b**), Ti31Mo5HA (**c**), Ti31Mo5HA1Ag (**d**), Ti31Mo5HA2CeO_2_ (**e**), Ti31Mo5HA2Ta_2_O_5_ (**f**).

**Figure 8:**
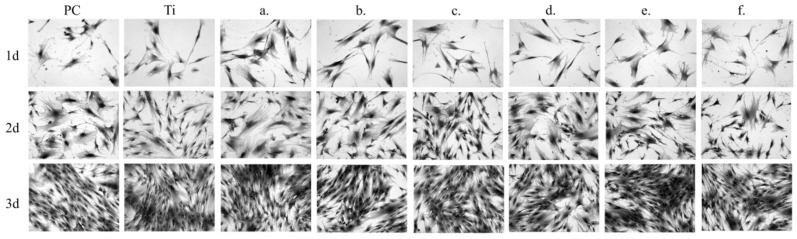
Morphology of the fibroblasts cells cultured for a different time: 24 h, 3 days, and 5 days: Ti31Mo (**a**), Ti31Mo after hydrothermal treatment (HT) (**b**), Ti31Mo5HA (**c**), Ti31Mo5HA1Ag (**d**), Ti31Mo5HA2CeO_2_ (**e**), Ti31Mo5HA2Ta_2_O_5_ (**f**).

The sample chemical composition and its microstructures as well as the time of culture of NHost and HPdLF influences strongly the final growth patterns. As we can see in Figure 7 and Figure 8, the growth rate was differentiated between the NHost and HPdLF cultures. Generally, NHost cells overgrow more regularly and faster on the tested Ti31Mo-type biomaterials.

**Figure 9:**
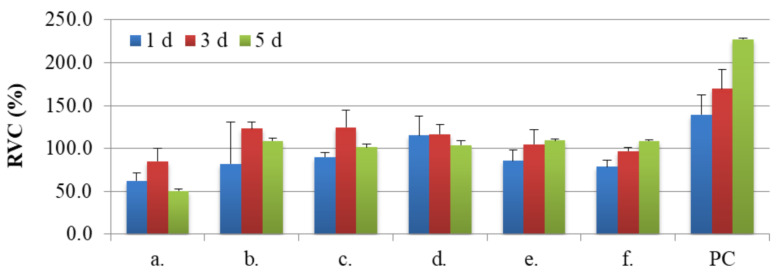
The results of the MTS assays performed at 1, 3, and 5 days on the viability of the osteoblasts for: Ti31Mo (**a**), Ti31Mo after hydrothermal treatment (**b**), Ti31Mo5HA (**c**), Ti31Mo5HA1Ag (**d**), Ti31Mo5HA2CeO_2_ (**e**), Ti31Mo5HA2Ta_2_O_5_ (**f**); PC—positive control.

**Figure 10:**
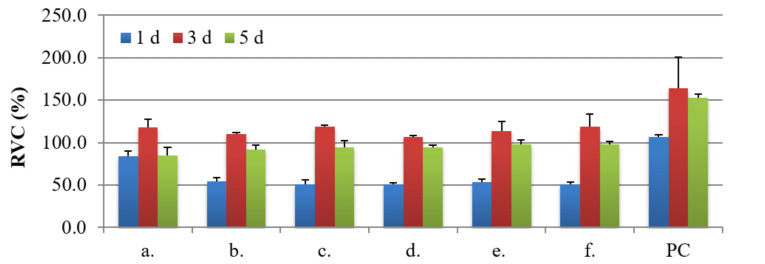
The results of the MTS assays performed at 1, 3, and 5 days on the viability of the fibroblasts for: Ti31Mo (**a**), Ti31Mo after hydrothermal treatment (**b**), Ti31Mo5HA (**c**), Ti31Mo5HA1Ag (**d**), Ti31Mo5HA2CeO_2_ (**e**), Ti31Mo5HA2Ta_2_O_5_ (**f**); PC—positive control.

Results of metabolic activity of NHost. and HPdLF and RVC values (%) for the reference sample (Ti) measured based on the MTS test after 24 h, 72 h, and 120 h of breeding in conditioned media are shown in Figure 9 and Figure 10; PC (positive control) is the cells of a given type bred in a fresh, unconditioned medium. Interesting results were noted for the Ti31Mo after hydrothermal treatment and for bulk Ti31Mo5HA1Ag, Ti31Mo5HA2CeO_2_, Ti31Mo5HA2Ta_2_O_5_ samples.

## 4. Discussion

β-type Ti alloys are interesting metallic materials for medical applications. Ultrafine-grained Ti-31 Mo alloy and Ti31Mo5HA, Ti31Mo5HA-Ag (or Ta_2_O_5_, CeO_2_) composites were synthesized and their properties investigated. The heat treatment of the amorphous material after the MA process led to the creation of β-type Ti31Mo alloy with a unique microstructure with a grain size of 2 μm. The increase of the HA concentration in the Ti31Mo composite increased the content of the α-phase. The alkali and hydrothermal treatment in the electrolyte containing 0.25M CaNa_2_-EDTA, 0.25M K_2_HPO_4_ in 1M NaOH at 120 °C for 2 h were applied. On a porous surface, the bioactive ceramic CaP layer was deposited.

In the corrosive environment of the tissue and body fluids, implants unexpected local corrosion. The corrosion products in the tissue can create a toxic effect [3]. The tests in the Ringer solution showed a positive effect on corrosion resistance of the CaP layer formed on ultrafine-grained Ti31Mo composite. This composite showed the best corrosion resistance after oxidation and CaP deposition (estimated weight loss of W = 0.015 mg/day). Contact angles of ultrafine-grained Ti31Mo alloy were determined in glycerol and show a visible decrease for bulk Ti31Mo alloy after oxidation and hydrothermal treatment (CA = 31°).

In vitro cytocompatibility of Ti-31 Mo alloy and Ti31Mo5HA, Ti31Mo5HA-Ag (or Ta_2_O_5_, CeO_2_) biocomposites was investigated and compared with commercial purity (CP) Ti. The cell lines of normal human osteoblasts (NHost) and human periodontal ligament fibroblasts (HPdLF) was conducted in the presence of tested biomaterials. NHost and HPdLF cells showed very good cell proliferation, colonization, and multilayering. The surface topography and the chemical composition of the biomaterial are key factors for the successful implant integration with the hard tissue. So, the biofunctionalization of synthesized composites represents an important procedure in the development of biomaterials that support the initial healing of the implant.

Silver has good antibacterial properties [54,56]. Earlier, the properties of Ti samples modified with nanodendrites of Ag were studied in detail [32]. These biomaterials have good biocompatibility. Recently, the antibacterial properties of Ti31Mo5HA composite containing Ag, Ta_2_O_5_, and CeO_2_ against *Staphylococcus aureus* was evaluated [18]. The Ti31Mo5HA1Ag and Ti31Mo5HA2CeO_2_ biomaterials have lower adhesion levels of *S. aureus* (*p* < 0.05). Additionally, these composites possess good mechanical properties [18]. Young’s modulus around 95 GPa is measured for bulk Ti31Mo5HA composites with 1 wt. % Ag and 2 wt. % CeO_2_ additions.

Good biocompatibility makes these biomaterials attractive in applications in implant applications. Performed in vitro studies confirm that ultrafine-grained bulk Ti31Mo-type composites altered with HA and Ag, Ta_2_O_5,_ or CeO_2_ did not show cytotoxic properties against cultured NHost and HPdLF cells. Independently, electrochemical anodic and cathodic surface treatment was applied to the Ti-6Zr-4Nb bulk alloy with nanostructure [57]. This treatment supports osteoblast adhesion and cell proliferation due to the created pores.

Recently, the properties of Ti-based scaffolds with a porosity of 70% and pore sizes in the range of 200–300 μm were synthesized by the application of titanium and ammonium hydrogen carbonate particles [58]. Anodization and heat treatment allows the formation of bioactive anatase nanotubes with the size of approximately 100 nm. Due to apatite creation, this surface modification on the Ti scaffold improved the biocompatibility. Finally, the compressive strength of 36.8 MPa was equal to the cancellous bone.

Until now, large numbers of new Ti-based alloys have been synthesized and their properties studied in vivo [12,13,14,20,21,22,23,24,25,26,44,45,46,49,50]. The environmental impacts and toxicity of ultrafine Ti-bioceramic composites should be evaluated. New implant biomaterials with β-crystal structure and ultrafine-grained microstructure should demonstrate a reduced susceptibility to bacterial colonization and should not have pathogenic effects. The ultrafine-grained Ti31Mo-type composites with the HA and Ag, Ta_2_O_5,_ or CeO_2_ addition may support the continuous adaptation process to the implant by the host organism.

## 5. Conclusions

In our study, bulk ultrafine-grained Ti31Mo-type composites with HA and Ag, CeO_2,_ or Ta_2_O_5_ additions were synthesized. The results of surface modifications of the ultrafine-grained Ti31Mo alloy were shown. This Ti31Mo alloy is favorable for biomedical applications. The modification of the alloy surface improves their properties. The alkali treatment (immersion in 5M NaOH (60 °C/24 h) and hydrothermal treatment in the electrolyte containing 0.25M CaNa_2_-EDTA, 0.25M K_2_HPO_4_ in 1M NaOH at 120 °C for 2 h, achieves promising results of surface fitting for implant applications. Ca-P layer formation during cathodic deposition is useful in osseointegration. The in vitro biocompatibility studies show that the bulk composites based on Ti31Mo5HA and Ag, CeO_2,_ or Ta_2_O_5_ are good candidates for future implant applications.

## Figures and Tables

**Table 1:** The structural parameters of Ti31Mo alloys before modification, after NaOH 60 °C/24 h, and hydrothermal treatment.

Structure Parameters			Ti31Mo	sTi31Mo (Ox)	Ti31Mo (HT)
**sig**		-	1.4629564	1.5791782	1.405559
**R**_**wp**_		[%]	6.0970262	6.329574	5.535124
**R**_**exp**_		[%]	4.167606	4.0081444	3.9381716
**Ti(β)**	A	[%]	**100.00**	**95.07**	-
	a	[Å]	3.2433(1)	3.2426(1)	-
	V	[Å^3^]	34.1(0)	33.5(0)	-
**Ti**_**3**_ **O**	A	[%]	-	**4.93**	-
	a	[Å]	-	5.1361(41)	-
	c	[Å]	-	9.5623(164)	-
	V	[Å^3^]	-	36.6(0)	-
**Ca**_**10**_**(PO**_**4**_)_**6**_**(OH)**_**2**_	A	[%]	-	-	**81.23**
	a	[Å]	-	-	9.4252(6)
	c	[Å]	-	-	6.8894(6)
	V	[Å^3^]	-	-	530.1(1)
**CaHPO**_**4**_ **·2H**_**2**_ **O**	A	[%]	-	-	**18.77**
	a	[Å]	-	-	6.3748(32)
	b	[Å]	-	-	15.2564(58)
	c	[Å]	-	-	5.7910(31)
	V	[Å^3^]	-	-	487.8(7)

**Table 2:** Energy-dispersive spectrometer (EDS) results for Ti31Mo after NaOH 60 °C/24 h and hydrothermal treatment.

Element	Line	Ti31Mo(Ox)	Ti31Mo(HT)
		wt. %	wt. %
Ti	Kα_1_	50.07	0.00
Mo	Lα_1_	41.63	0.00
Ca	Kα_1_	0.00	45.07
P	Kα_1_	0.00	24.28
O	Kα_1_	6.07	16.56
Na	Kα_1_	2.22	6.81
K	Kα_1_	0.00	7.28
**Total**	**-**	**100.00**	**100.00**

**Table 3:** Two-dimensional (R_a_, R_t_, R_z_) parameters for the Ti31Mo alloy before modification, after NaOH 60 °C/24 h, and hydrothermal treatment; parameters are taken from the surface area of 1.08 mm^2^.

2D Parameters	Ti31Mo	Ti31Mo (Ox)	Ti31Mo (HT)
**R**_**a**_	1.04 ± 0.13	2.40 ± 0.36	9.22 ± 1.93
**R**_**t**_	14.55 ± 0.78	22.18 ± 1.85	62.52 ± 10.85
**R**_**z**_	10.59 ± 0.77	15.70 ± 1.69	45.39 ± 5.95

**Table 4:** Estimated weight loss (W) after 7-days immersing in Ringer solution environment and contact angle (CA), surface free energy, disperse, and polar for Ti31Mo before modification, after NaOH 60 °C/24 h and hydrothermal treatment.

Parameter	Unit	Ti31Mo	Ti31Mo (Ox)	Ti31Mo (HT)
**W**	**(mg/day)**	0.0496(16)	0.0357(22)	0.0150(25)
**Diiodomethane CA**	**(°)**	58.76 ± 4.54	46.16 ± 5.71	impossible to measure
**Glycerol CA**	**(°)**	50.12 ± 1.39	55.97 ± 5.31	31.28 ± 2.78
**Surface free energy**	**(mN/m)**	43.16 ± 0.37	42.54 ± 3.07	-
**Disperse**	**(mN/m)**	29.29 ± 2.61	36.37 ± 3.10	-
**Polar**	**(mN/m)**	13.87 ± 2.87	6.17 ± 1.41	-
